# Effects of individualized PEEP on postoperative delirium among older patients in prone position: study protocol for a prospective randomized controlled trial

**DOI:** 10.1186/s12883-022-02990-x

**Published:** 2022-12-09

**Authors:** Wenchao Zhang, Shuang Han, Jianli Li

**Affiliations:** 1 Department of Anesthesiology, Hebei General Hospital, 050051 Shijiazhuang, Hebei Province China; 2 Graduate Faculty, Hebei North University, 075000 Zhangjiakou, Hebei Province China

**Keywords:** Postoperative Delirium, Prone Position, Individualized PEEP, Ventilation, Aged

## Abstract

**Background:**

Postoperative delirium (POD) is an acute mental disorder that often occurs in the elderly after surgery. It can delay functional recovery, extend hospital stay, and increase 1-year mortality and financial costs. Studies have shown that inflammation and cerebral desaturation are the potential pathogenesis of postoperative delirium. Prone-position surgery increases peak airway pressure and decreases lung compliance, exacerbating ventilator-induced inflammation response, as well as the decrease of the patient's cerebral oxygen saturation. Recent studies demonstrated that lung-protective ventilation strategy could reduce inflammatory response and improve cerebral oxygen saturation (rSO_2_) to prevent POD. In this trial, we aim to investigate the effect of the individualized PEEP on postoperative delirium in elderly patients during prone position.

**Methods:**

A prospective, randomized clinical trial will be performed in Hebei General Hospital. 106 patients undergoing elective surgery in prone position will be randomly divided into controlled group (group C) and individualized PEEP group (group I). Lung-protective ventilation with tidal volume of 6ml/kg predictive body weight (PBW) and positive end-expiratory pressure (PEEP) of 5 cm H_2_O will be applied to patients in group C. Patients in group I will receive the same tidal volume as described in group C plus individualized PEEP corresponding to the maximum dynamic compliance (Cdyn) in the titration method. Our primary endpoint is the prevalence of postoperative delirium based on the Confusion Assessment Method (CAM) until postoperative day 3. Secondary endpoints include the intraoperative rSO_2_, respiratory variables, arterial blood gases, lung ultrasound score (LUS), postoperative VAS score, and plasma concentrations of IL-6, IL-1β and neuron-specific enolase (NSE).

**Discussion:**

The results of the current protocol might provide evidence for individualized PEEP to prevent POD among older surgical patients in prone position.

**Trial registration:**

Chinese Clinical Trial Registry (ChiCTR2200056001). Registered 2022 January 30, http://www.chictr.org.cn/index.aspx.

## Background

Postoperative delirium, a serious postoperative neurological complication, characterized by acute and fluctuating changes in consciousness and attention, can increase morbidity and mortality in patients over 60 years of age [1]. Due to different types of surgery, POD in elderly patients has been reported to occur in 10%-60% [2].

Mechanical ventilation could induce proinflammatory response and ventilator-induced lung injury (VILI), which may increase the incidence of POD [3, 4]. The prone position is commonly used during percutaneous nephrolithotripsy or spine surgery to provide a fine condition for the operation. However, this non-physiological position can cause increased peak airway pressure (P_peak_), decreased Cdyn, thus aggravating lung injury [5, 6]. In addition, perioperative cerebral desaturation may increase the risk of POD among different types of surgery [7, 8]. Significantly, cerebral desaturation commonly occurs in elderly surgical patients undergoing prone position [9]. Thus, patients undergoing prone position may be at high risk of POD owing to decreased rSO_2_ and VILI-induced inflammatory response. Recent research suggested that intraoperative lung-protective ventilation might help prevent POD by inhibiting inflammation and improving rSO_2 _[4].

Intraoperative lung-protective ventilation strategies, including low tidal volumes (V_T_), PEEP, and recruitment maneuvers (RM), have been proved to alleviate lung injury and prevent POD [4, 10]. Our previous studies have shown that individualized PEEP combined with pressure-controlled ventilation-volume guaranteed (PCV-VG) mode could mitigate lung damage, and lead to favorable oxygenation in patients undergoing thoracoscopic surgery and laparoscopic surgery [11, 12]. Recent studies demonstrated that the application of PEEP could induce significant increases in rSO_2_ [4, 13]. Whether individualized PEEP could prevent POD is not clear. Hence, we hypothesized that individualized PEEP can prevent POD by reducing inflammatory response and improving rSO_2_.

Based on the above studies, our aim is to explore the neuroprotective effect of individualized PEEP among elderly patients undergoing mechanical ventilation in prone position. This trial will replenish further clinical evidence of employing intraoperative individualized PEEP to prevent POD.

## Methods

This study has been approved by the Ethics Committee for Clinical Trial of Hebei General Hospital, China (no.2019–48) and has been registered in the Chinese Clinical Trial Registry (registration number ChiCTR2200056001).

### Trial design

**Fig. 1:**
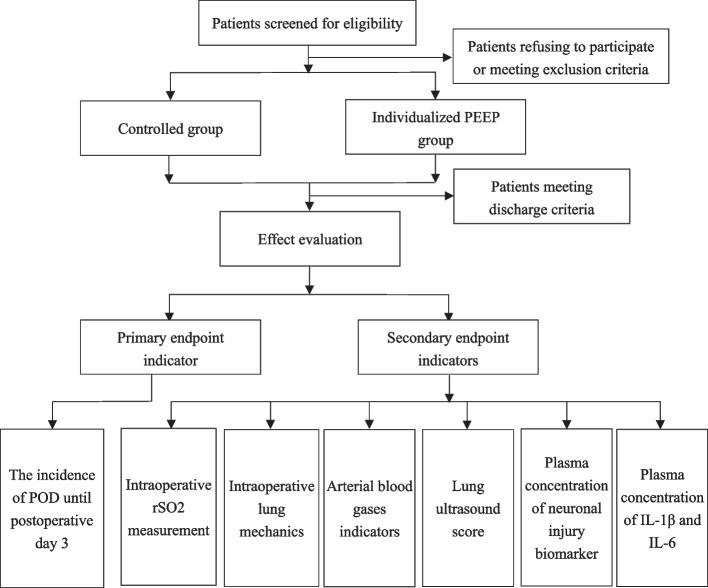
Flow chart of the trial

This single-center, prospective, randomized controlled trial is designed to explore whether individualized PEEP application would prevent POD in elderly surgical patients in prone position. Figure 1 shows the Flow chart of the trial. This study will be performed at Hebei General Hospital according to the standard protocol items for randomized trials [14]. The investigators will collect data from the day before surgery to the time the patients are discharged from the hospital (Table 1).

**Table 1.**
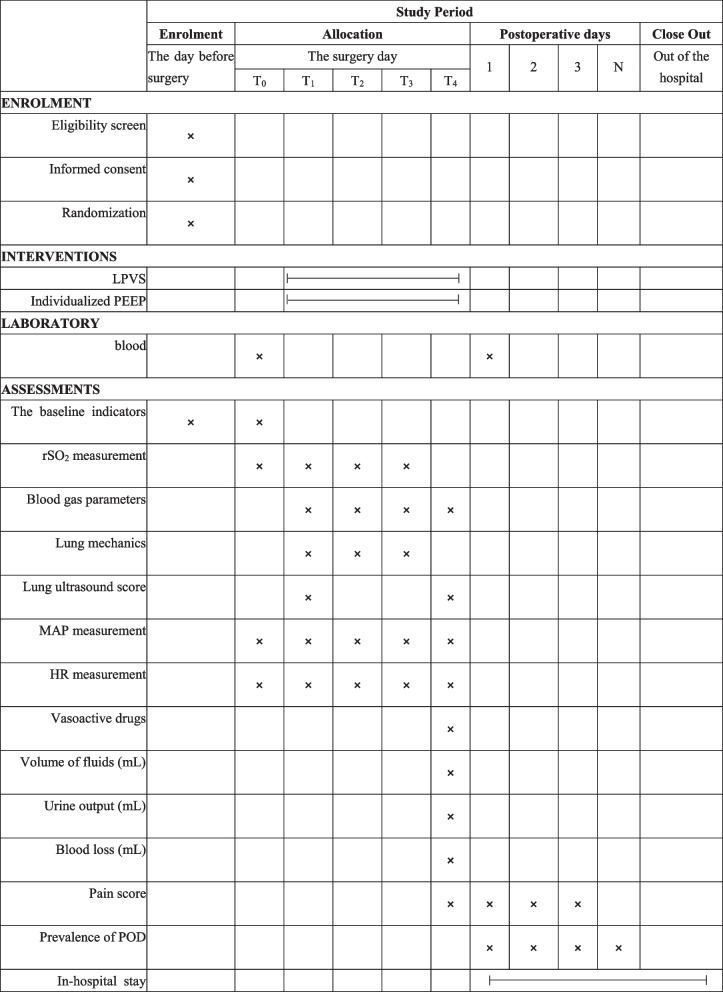
Standard Protocol Items: Recommendations for Interventional Trials (SPIRIT) Schedule for enrollment, interventions, and assessments. T0: after entering the operating room, T1: 5 minutes after starting mechanical ventilation in the supine position, T2: 60 minutes after turning over, and T3: 5 minutes after taking in the supine position, T4: after breathing air in PACU for 20 minute

### Randomization and blinding

The trial is single-blind because the attending anesthesiologist will collect data and perform PEEP titration without being blinded to the group assignments. Based on a computer-generated randomization sequence performed by an independent investigator, the selected participants will be randomized into Group I and Group C. Envelopes with random numbers will be opened when patients enter the operating room. The allocation ratio is 1:1. Postoperative information collection and the following data analyses will be performed by another researcher who will not participate in this study and know about the grouping allocation.

### Study participants and recruitment

In this clinical trial, 106 patients scheduled to undergo elective surgeries in prone position will be recruited from February 1, 2022 to February 1, 2023. Participants will receive written information about the study and provide written informed consent. We will enroll patients who meet the inclusion criteria and sign informed consent one day before surgery.

### Inclusion criteria

Patients who plan to undergo elective surgery in prone positions will be included according to the criteria below: (1) ASA physical status II and III; (2) ≥ 65 years old; (3) baseline mini-mental state examination (MMSE) [15] score > 23 points evaluated one day before surgery.

### Exclusion criteria

Patients who meet the following criteria will be excluded: (1) hypoalbuminemia or hypoxemia; (2) anemia; (3) chronic lung disease; (4) mental illness; (5) BMI ≥ 35 kg/m^2^; (6) contraindications to PEEP [16] (right heart failure, high intracranial pressure, or hypovolemic shock).

### Discharge criteria

The discharge criteria are as follows: (1) baseline rSO2 < 60%; (2) the operation time > 4 h, (3) intraoperative blood loss > 800 ml; (4) interruption of study protocol.

### Interventions

Noninvasive blood pressure (NIBP), electrocardiogram, pulse oxygen saturation, bispectral index (BIS), body temperature, and rSO_2_ of patients in each group will be routinely monitored after entering the operating room. We will place two brain oxygen electrodes 4 cm from the forehead to the brow arch (one on the left and another on the right) to measure rSO_2_ by using near-infrared spectroscopy (NIRS). After placement of monitors, radial artery puncture and catheterization will be performed under local anesthesia, allowing intermittent blood gas analyses and continuous hemodynamic monitoring.

Patients will receive intravenous propofol (2 mg/kg), sufentanil (0.3 µg/kg), and cisatracurium (0.15 mg/kg) for anesthesia induction and tracheal intubation after preoxygenated (inspired oxygen fraction of 0.8) for three minutes. During surgery, sevoflurane inhalation and continuous intravenous remifentanil and propofol infusion will be employed to maintain BIS between 40 and 60. Intermittent administration of cis-atracurium will provide sufficient muscle relaxation. Vasoactive drugs will be used to maintain mean arterial pressure (MAP) and heart rate within ± 20% of the baseline. The core temperature will be maintained above 36.0 ℃ by using a forced air warming system. Subsequently, when patients can respond to verbal commands, they will undergo extubating procedures and be transferred to the postanesthesia care unit (PACU) until fully awake. Patients will routinely receive intravenous patient-controlled analgesia (PCIA) to control postoperative pain.

Mechanical ventilation will be maintained with V_T_ 6 ml/kg PBW, FIO_2_ of 50%, and I: E of 1:2 with PCV-VG mode. After the prone position is established, both groups will receive the recruitment maneuver by squeezing a reservoir bag up to 30–40 cm H_2_O for 15–20 s. The initial respiratory rate will be set to 12 breaths per minute and P_ET_CO_2_ is maintained in the range of 40 ± 5 mm Hg by adjusting the respiration rate.

**Fig. 2:**
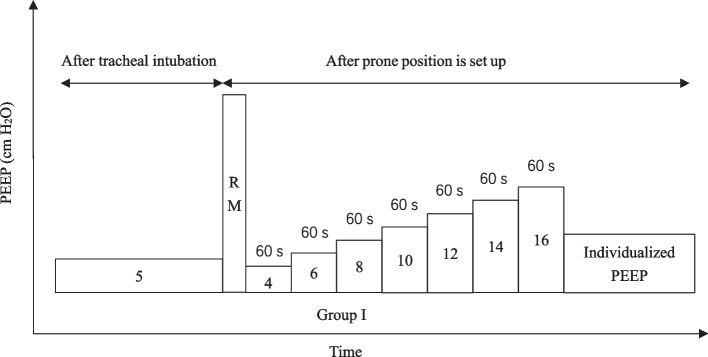
Study protocol of the incremental PEEP titration procedure determined by Cdyn in patients of the Group I

The patients in both groups will receive the PEEP of 5 cm H_2_O after endotracheal intubation, and this PEEP level will be maintained throughout the whole procedure in the group C. After the prone position is set up, patients in Group I will receive optimal individualized PEEP identified by an incremental PEEP titration protocol (Fig. 2). The PEEP titration method is as follows: each PEEP level (4, 6, 8, 10, 12, 14, 16 cm H_2_O) will be maintained for 60 s. Meanwhile, we will record the Cdyn at each PEEP level. We will select the optimal PEEP level based on the maximum Cdyn and maintain it throughout the whole study period. If circulatory instability occurs during PEEP titration, intravenous infusion of noradrenaline will be used to maintain MAP ≥ 55mmHg.

### Outcome assessment

The primary endpoint of this trial will be the incidence of POD within 3 days after surgery. POD will be determined by an independent researcher who will not know the group assignment using CAM [17]. The investigator will participate in a training session including symptoms and diagnosis of delirium and will not know the group assignment. The researcher will assess POD once daily, between 6: 00 am to 8: 00 am. Four key delirium characteristics consist of CAM diagnostic criteria: (1) acute onset and disease fluctuations, (2) inattention, (3) changes in consciousness, and (4) disordered thinking. Delirium will be defined as the presence of (1) and (2), accompanied by (3) or (4) or both [7].

The secondary outcomes will include perioperative rSO_2_ measurement, arterial blood gases, respiratory variables, and plasma concentrations of interleukin (IL)-1β, IL-6, and NSE. The baseline rSO_2_ will be obtained within 2 minutes before anesthesia induction and averaged. Additionally, intraoperative rSO_2_ values will be continuously measured and recorded using a cerebral oximeter at the following timepoint: T_0_: after entering the operating room; T_1_: 5 minutes after starting mechanical ventilation in the supine position; T_2_: 60 minutes after turning over; and T_3_: 5 minutes after taking in the supine position. The serum concentrations of interleukin (IL)-1β, IL-6, and NSE will be determined by ELISA kits. We will collect the blood samples 15 minutes before anesthesia induction (T_0_) and postoperative day 1.

Arterial blood gases will be measured at T_1_ to T_4_ (after breathing air in PACU for 20 minutes). Respiratory variables including P_ET_CO_2_, PEEP, P_peak_, mean airway pressure (P_mean_), Cdyn, PaO_2_/FiO_2_, A-aDO_2_, V_D_/V_T,_ and Qs/Qt will be recorded at T_1_ to T_3_. Each patient will receive two ultrasound examinations at T_1_ and T_4_. And lung ultrasound scans will be performed by an experienced anesthetist who will not know the group allocations. As described previously [18, 19], each hemithorax will be alienated into 6 quadrants and each region will be assigned a score of 0 to 3 according to the different signs of B-line, subpleural consolidation and pleural line under ultrasound scan. The LUS will be calculated to quantitatively assess the aeration loss by adding all scores up.

Postoperative VAS scores will be checked by the ward doctors who will not be involved in this study. When the VAS score is>3 points, participants will receive intravenous Flubiprofen infusion with the dose of 50 mg. The analgesic drug consumption will also be recorded.

Parameters calculating will use formulas below:

$${\mathrm V}_{\mathrm D}/{\mathrm V}_{\mathrm T}=\;(\mathrm{PaCO}2-\;{\mathrm P}_{\mathrm{ET}}{\mathrm{CO}}_2)/{\mathrm{PaCO}}_2$$

$$\mathrm{Qs}/\mathrm{Qt}\;=\;(\mathrm{PA}-{\mathrm{aDO}}_2\times0.0031)\;\div\;(\mathrm{PA}-{\mathrm{DO}}_2\times\;0.0031\;+\;5)$$

$$\mathrm{PA}-\mathrm{aDO}2=\;\lbrack{\mathrm{FiO}}_2\times\;({\mathrm P}_{\mathrm B}-\;{\mathrm P}_{\mathrm H2\mathrm O})\rbrack\;-\;{\mathrm{PaCO}}_2/\mathrm R\;-\;\mathrm{PaO}2\;$$

$$(\mathrm{PB}=\;760\mathrm{mmHg},\;{\mathrm P}_{\mathrm H2\mathrm O}=\;47\mathrm{mmHg},\;\mathrm R\;=\;0.8)$$

### Sample size calculation

G∗power (version 3.1.9.6) is used to calculate the sample size. A total of 16 (8 in the I group and 8 in the C group) patients were included in our preliminary experiments. The number of patients diagnosed with POD was 2 in the I group and 3 in the C group, respectively. On this basis, the calculated sample size that would provide 80% power to detect this difference at a two-sided significance level of 0.05 was 42 patients per group. Considering a dropout rate of 20%, 106 patients will be enrolled in this study.

### Statistical methods

SPSS (version 22.0, IBM) will be used for statistical analyses. The data with a normal distribution are expressed as mean ± standard deviation (SD) and compared by independent-samples t tests. The data of non-normally distribution are expressed as median (interquartile range) and compared by Mann-Whitney U-tests. Categorical data are described as numbers or percentages and analyzed using Fisher’s exact test or chi-squared test. Univariate repeated measures ANOVA will be used for intra-group comparisons and post hoc tests with Bonferroni correction will be used to control for Type I error. We will perform a Mantel–Haenszel test, stratified according to whether MAP < 55 mmHg occurs during PEEP titration and other possible confounding factors, to compare the risks of POD in each group. *P* values < 0.05 will be considered statistically significant.

### Protocol amendments

The ethical committee will review all changes about the study protocol, which will be reported to the sponsor and investigators.

### Data monitoring

The data monitoring committee (DMC) consists of an independent researcher, who responsible for data collection and classification, and a statistician. The DMC will only perform data monitoring and not be included in the study. The baseline characteristics of individuals enrolled in the groups will be recorded. Monitoring will verify the completeness and accuracy of the data and assess the progress of the research. At the end of the trial, we will submit the original data and the final experimental dataset and consequences to the scientific research management committee.

### Safety evaluation

The researchers will monitor all adverse events (AEs) associated with the PEEP titration procedure. AEs are defined as hypotension, significant cardiac arrhythmias, pneumothorax, significant disability or incapacity, and even death. The researchers will visit all patients in the trial daily until they are discharged from the hospital and record the occurrence of all AEs in detail. Once AEs occur, the trial will be suspended and an investigation will begin.

## Discussion

POD is a state of acute brain dysfunction, characterized by fluctuating alterations of consciousness, attention, and cognition [20]. As a common postoperative complication of hospitalized elderly patients, POD usually occurs 2–5 days after surgery and it is related to prolonged length of hospital stay and decreased functional independence [2, 21]. Additionally, POD could result in long-lasting cognitive dysfunction even in recovered patients [22]. Therefore, it is of important clinical significance to explore the pathogenesis and prevention measures of POD in elderly patients to prevent POD and promote recovery.

Patients undergoing prone position surgery such as percutaneous nephrolithotripsy or posterior spinal surgery are prone to deterioration of airway pressure, pulmonary compliance, and rSO_2 _[9]. The rSO_2_, an indicator of brain oxygen consumption and supplementation, can predict brain hypoxia [23]. Two main factors may lead to the decrease in rSO_2_ when position changes from supine to prone position [9, 24]. Firstly, caval compression may lead to a reduced cardiac output, which can decrease the perfusion pressure of the brain. Alternatively, the increase of intrathoracic pressure may affect the reflux of cerebral venous blood volume to the right atrium, resulting in an increase in the proportion of cerebral venous blood volume and a decrease in rSO_2_. Previous study has shown that the reduction of intraoperative rSO_2_ may increase the risk of POD [25]. The mechanism may be that reduced oxygen supply to the brain leads to hypoxia and metabolic dysfunction of brain cells, thereby reducing the number of neurons and leading to POD [4].

Inflammation is another major risk factor for POD [26]. Mechanical ventilation could account for VILI and might induce the release of proinflammatory mediators. Due to the decrease of lung compliance in elderly patients, inflammation and VILI are more likely to occur during mechanical ventilation. However, peripheral inflammatory factors can exacerbate the inflammation of the brain and contribute to neuronal injury [27]. Proinflammatory markers such as IL-1β and IL-6 could trigger neuroinflammation and may be associated with cognitive decline [28, 29]. Surgical patients undergoing prone positions tend to have increased airway pressure and decreased pulmonary compliance, which could lead to inflammation response and lung injury [4]. Therefore, appropriate ventilation strategies may be useful to prevent VILI and POD.

A recent study has shown that POD can be prevented by employing intraoperative lung-protective ventilation consisting of V_T_ of 6 ml/kg PBW and PEEP of 5 cm H_2_O in elderly patients undergoing posterior spinal surgery in prone position[4]. Lung-protective ventilation, consisting of a low tidal volume, recruitment maneuvers, and moderate PEEP, has been found to effectively decrease the incidence of PPCs [30]. Meanwhile, lung-protective ventilation is an appropriate way of reducing the systemic proinflammatory response because of the use of a moderate PEEP and the reduction of tidal volume [31]. Previous studies demonstrated that the application of PEEP could improve rSO_2 _[4, 13]. However, applying a low V_T_ with a fixed or insufficient PEEP for all patients is inappropriate because this may lead to atelectrauma [32]. Our team found that employing individualized PEEP can improve oxygenation and reduce inflammatory markers compared with fixed PEEP strategies in patients undergoing thoracoscopic and laparoscopic surgery [11, 12]. Hence, we assume that individualized PEEP can prevent POD among elderly surgical patients in prone position by increasing rSO_2_ and reducing inflammation.

This study has two main limitations. First, all patients will be recruited from a single center. Second, the sample size of this trial is limited and a larger clinical trial is needed. To sum up, the trial is hopefully to replenish new clinical evidence for individualized PEEP to prevent POD in surgical elderly patients undergoing prone position.

### Trial status

So far, we have recruited 12 participants, and the trial has not been completed participant recruitment at the time of submission. The study will begin in January 2022 and the recruitment phase will last for one year. Protocol version 1.0.

## Data Availability

The datasets used and/or analyzed during the current study are available from the corresponding author on reasonable request.

## Abbreviations

AEs: adverse events
BIS: bispectral index
CAM: Confusion Assessment Method
Cdyn: dynamic compliance
FIO_2_: fraction of inspired oxygen
LUS: lung ultrasound score
MAP: mean arterial pressure
NIBP: Noninvasive blood pressure
NIRS: near-infrared spectroscopy
NSE: neuron-specific enolase
PACU: postanesthesia care unit
PBW: Predictive body weight
PCIA: intravenous patient-controlled analgesia
PCV-VG: pressure‑controlled ventilation‑volume guaranteed
PEEP: Positive end-expiratory pressure
P_ET_CO_2_: end-tidal carbon dioxide
P_mean_: mean airway pressure
POD: Postoperative delirium
P_peak_: Peak airway pressure
RM: Recruitment maneuvers
rSO_2_: Cerebral oxygen saturation
VILI: Ventilator-induced lung injury
V_T_: tidal volumes
